# Dietary Mannan Oligosaccharides Enhance Lactational Performance, Nutrient Metabolism, Plasma Metabolomics, and Gut Microbiota in Dezhou Donkeys

**DOI:** 10.3390/ijms26189105

**Published:** 2025-09-18

**Authors:** Tianzheng Wang, Yaru Wang, Pengshuai Li, Jiaxin Liu, Xinyi Mao, Zuowei Li, Zhangxinhan Wen, Yuhan Yin, Yan Li, Gang Lin, Haihua Zhang, Honglei Qu, Qiugang Ma, Shimeng Huang

**Affiliations:** 1 State Key Laboratory of Animal Nutrition, College of Animal Science and Technology, China Agricultural University, Beijing 100193, China; xiaogenishi@163.com (T.W.); wyrsmile0930@163.com (Y.W.); lpsmyj@cau.edu.cn (P.L.); jxliu@cau.edu.cn (J.L.); mxycindy0929@163.com (X.M.); 15684302290@163.com (Z.L.); zl_one@163.com (Z.W.); vvvirrgil@outlook.com (Y.Y.); leihong_qu@163.com (H.Q.); maqiugang@cau.edu.cn (Q.M.); 2 Hebei Key Laboratory of Specialty Animal Germplasm Resources Exploration and Innovation, College of Animal Science and Technology, Hebei Normal University of Science and Technology, Qinhuangdao 066004, China; zhh83@126.com; 3 College of Animal Sciences, Xinjiang Agricultural University, Urumqi 830052, China; yanlee@xjau.edu.cn; 4 Institute of Quality Standards and Testing Technology for Agricultural Products, Chinese Academy of Agricultural Science, Beijing 100081, China; lingang@caas.cn

**Keywords:** mannan oligosaccharides, Dezhou donkey, lactation, gut microbiota, serum metabolome

## Abstract

This study investigated the effects of dietary mannan oligosaccharide (MOS) supplementation on growth performance, serum biochemistry, metabolomic profiles, and fecal microbiota in lactating Dezhou donkeys. Sixteen healthy jennies and their foals were randomly allocated to a control group (MCON), a group receiving no MOS, or an MOS-supplemented group (MMO; 0.5 g/kg diet) for 60 days. Compared with the MCON group, the MMO group showed a mitigation of lactational weight reduction, improved serum protein profiles, and favorable modulation of lipid metabolism. Furthermore, serum metabolomic analysis revealed 102 differentially abundant metabolites, which were enriched in 17 KEGG pathways involved in energy metabolism, bile secretion, and anti-inflammatory signaling. Key metabolites such as L-4-Chlorotryptophan, Gly-Trp, and cholylthreonine indicated enhanced nutrient metabolism and gut barrier function. Moreover, MOS supplementation significantly increased alpha diversity of the gut microbiota, altered community composition, and promoted the abundance of beneficial genera, including Clostridium and Bacteroides. Collectively, these results demonstrate that MOS supplementation improves metabolic health, modulates immune and antioxidant responses, and fosters a beneficial gut microbial ecosystem in lactating donkeys, suggesting its potential as an effective prebiotic in equine nutrition.

## 1. Introduction

Mannan oligosaccharides (MOSs), primarily derived from the outer cell wall of Saccharomyces cerevisiae, are non-digestible prebiotics known for their diverse roles in improving animal health and productivity. Structurally, MOS contains α-1,2, α-1,3, and α-1,6 glycosidic bonds [1], which provide high stability during feed processing and in the gastrointestinal tract, making it a practical dietary supplement in livestock production [2]. Functionally, MOS modulates gut microbiota, enhances immune responses, improves antioxidant capacity, and regulates metabolic pathways, establishing it as a sustainable alternative to antibiotics. For instance, in swine, MOS supplementation (0.2%) elevated the CD4^+^/CD8^+^ T-cell ratio to near-optimal levels (1.86 vs. 1.47), indicating strengthened cellular immunity [3]. Similarly, in weaned piglets, MOS (0.2%) increased average daily gain by 14% and reduced diarrhea incidence by 32.7%, accompanied by improved intestinal morphology, including greater villus height (2.313 μm vs. 1.870 μm) and optimized crypt structure [2]. Moreover, MOS reinforces intestinal barrier function by stimulating mucus secretion, maintaining epithelial integrity, and reducing inflammatory susceptibility [4]. Additionally, it suppresses pro-inflammatory cytokines such as IL-1β, IL-6, and IL-8 while activating anti-inflammatory pathways [5]. In murine colitis models, konjac-derived MOS alleviated inflammation through macrophage activation mediated by the mannose receptor SIGNR1 [6]. Given these documented benefits across species, MOS represents a promising prebiotic for enhancing health and productivity in lactating donkeys. However, its efficacy and mechanistic actions in donkeys remain poorly characterized. Therefore, further studies are essential to elucidate the functional roles of MOS in this species, which will support the development of evidence-based nutritional strategies and contribute to the sustainable advancement of the donkey industry.

As a historically important domesticated species, the donkey has provided agricultural labor, hides, and dairy products for centuries [7]. The Dezhou donkey, one of China’s five major native breeds and mainly distributed in Shandong Province, is known for its robust build, adaptability, roughage tolerance, and disease resistance [8]. Donkey milk has also gained interest due to its nutritional similarity to human milk, especially in lactose, protein, and fat content, making it suitable for food and pharmaceutical uses [9]. With the expansion of the donkey industry and rising restrictions on antibiotics, there is a growing demand for safe and sustainable feed additives that support animal health and production [9,10]. Mannan oligosaccharides (MOS), a prebiotic derived from yeast, have shown promise in improving gut health, immunity, and metabolism in several livestock species, offering a viable alternative to antibiotics. However, despite these benefits in other animals, the effects of MOS on donkey metabolism, gut microbiota, and lactation performance remain unclear. Therefore, investigating the role of MOS in lactating jennies is crucial to developing evidence-based feeding strategies and promoting sustainable donkey production.

Accordingly, this study was designed to evaluate the effects of dietary MOS supplementation on body weight change, serum biochemical parameters, plasma metabolome, and fecal microbiota in lactating Dezhou donkeys. We hypothesized that MOS would enhance metabolic health, improve gut function, and reduce lactation-induced physiological stress, thereby contributing to improved lactational performance and overall health in jennies.

## 2. Results

### 2.1. Effects of Mannan Oligosaccharides on Growth Performance in Dezhou Donkeys

**Figure 1:**
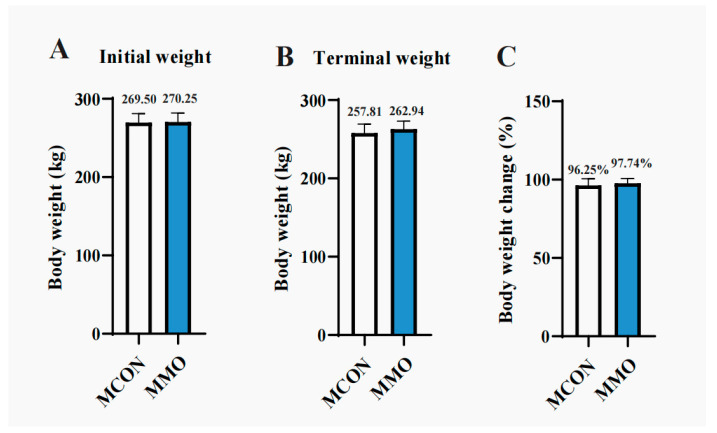
Effects of Mannan Oligosaccharides on Growth Performance in Dezhou Donkeys: MCON served as the non-MOS-supplemented group. (**A**) Initial body weight; (**B**) Final body weight; (**C**) Percentage change in body weight. Data are presented as mean ± standard deviation (*n* = 8 per group).

By the end of the trial, the MMO group showed a numerically reduced body weight loss during lactation relative to the MCON group, though the difference was not statistically supported (*p* > 0.05), as presented in Figure 1.

### 2.2. Effects of Mannan Oligosaccharides on Blood Biochemical Parameters in Dezhou Donkeys

**Figure 2:**
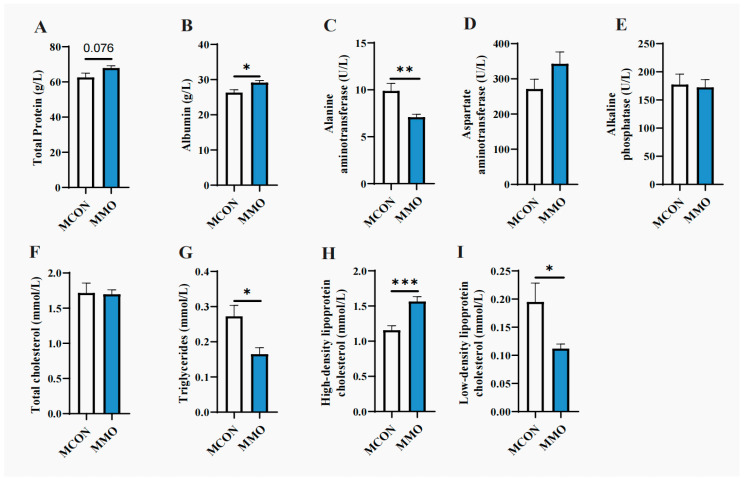
Effects of Mannan Oligosaccharides on Blood Biochemical Parameters in Dezhou Donkeys. (**A**) Total Protein (g/L); (**B**) Albumin (g/L); (**C**) Alanine aminotransferase (U/L); (**D**) Aspartate aminotransferase (U/L); (**E**) Alkaline phosphatase (U/L); (**F**) Total cholesterol (mmol/L); (**G**) Triglycerides (mmol/L); (**H**) High-density lipoproteincholesterol (mmol/L); (**I**) Low-density lipoproteincholesterol (mmol/L). Data are presented as mean ± standard deviation (*n* = 8 per group). * *p* < 0.05, ** *p* < 0.01, *** *p* < 0.001.

Serum TP and ALB levels were numerically higher in the MMO group than in the MCON group, with the difference in ALB being statistically significant (*p* < 0.05). Liver function markers also showed notable changes: ALT was significantly reduced (*p* < 0.01), and ALP activity exhibited a downward trend. In terms of lipid metabolism, both TC and TG were numerically lower—with the reduction in TG reaching significance (*p* < 0.05)—while HDL-C was strongly elevated (*p* < 0.001), and LDL-C was significantly decreased (*p* < 0.05) in the MMO group relative to the MCON group (Figure 2).

### 2.3. Effects of Mannan Oligosaccharides on Serum Metabolome in Dezhou Donkeys

**Figure 3:**
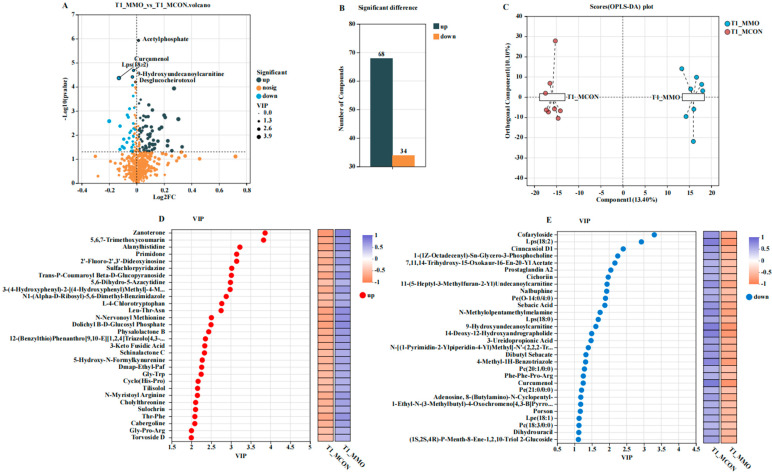
Effects of Mannan Oligosaccharides on Serum Metabolome in Dezhou Donkeys. (**A**) Volcano plot of differential metabolites between the MCON and MMO groups; (**B**) Bar plot of different metabolites between the MCON and MMO groups; (**C**) OPLS-DA score plot; (**D**) VIP score plot of upregulated differential metabolites in the MMO group; (**E**) VIP score plot of downregulated differential metabolites in the MMO group. Data are presented as mean ± standard deviation (*n* = 8 per group).

**Figure 4:**
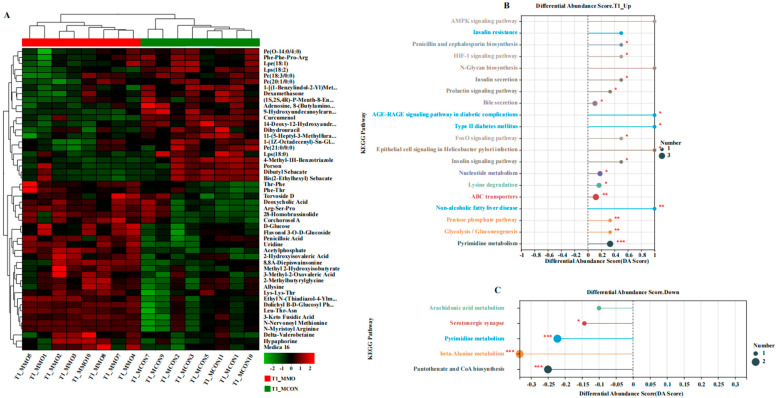
Effects of Mannan Oligosaccharides on Functional Pathways of Differential Serum Metabolites in Dezhou Donkeys. (**A**) Heatmap of upregulated and downregulated differential serum metabolites; (**B**) KEGG pathway enrichment analysis of upregulated differential serum metabolites in the MMO group; (**C**) KEGG pathway enrichment analysis of downregulated differential serum metabolites in the MMO group. Data are presented as mean ± standard deviation (*n* = 8 per group). * *p* < 0.05, ** *p* < 0.01, *** *p* < 0.001.

A total of 102 differential metabolites were identified (VIP > 1, *p* < 0.05), with 68 upregulated and 34 downregulated in the MMO group. KEGG enrichment analysis revealed 17 metabolic pathways with notable alterations (*p* < 0.05). Upregulated pathways were primarily related to energy metabolism (e.g., glycolysis/gluconeogenesis, pentose phosphate pathway), bile secretion, and anti-inflammatory signaling (e.g., FoxO signaling, AGE-RAGE signaling). Downregulated pathways included serotonergic synapse, beta-alanine metabolism, and pantothenate and CoA biosynthesis (Figure 3 and Figure 4). Key upregulated metabolites such as L-4-Chlorotryptophan, Gly-Trp, and cholylthreonine are associated with improved nutrient metabolism, antioxidant capacity, and gut barrier integrity, whereas prostaglandin A2 was downregulated.

To further interpret the biological relevance of the observed metabolic changes, KEGG pathway enrichment analysis was conducted. A total of 17 pathways showed clear upregulation in the MOS group (all *p* < 0.05 or *p* < 0.01), encompassing bile secretion, insulin secretion and signaling, FoxO signaling, HIF-1 signaling, AGE-RAGE signaling, prolactin signaling, ABC transporters, glycolysis/gluconeogenesis, pentose phosphate pathway, and nucleotide metabolism. These are functionally linked to glucose and lipid metabolism, bile acid transport, energy production, oxidative stress regulation, and endocrine modulation. Conversely, pathways including serotonergic synapse, beta-alanine metabolism, and pantothenate and CoA biosynthesis were downregulated (*p* < 0.05 or *p* < 0.001).

### 2.4. Effects of Mannan Oligosaccharide Supplementation on the Fecal Microbial Community in Dezhou Donkeys

**Figure 5:**
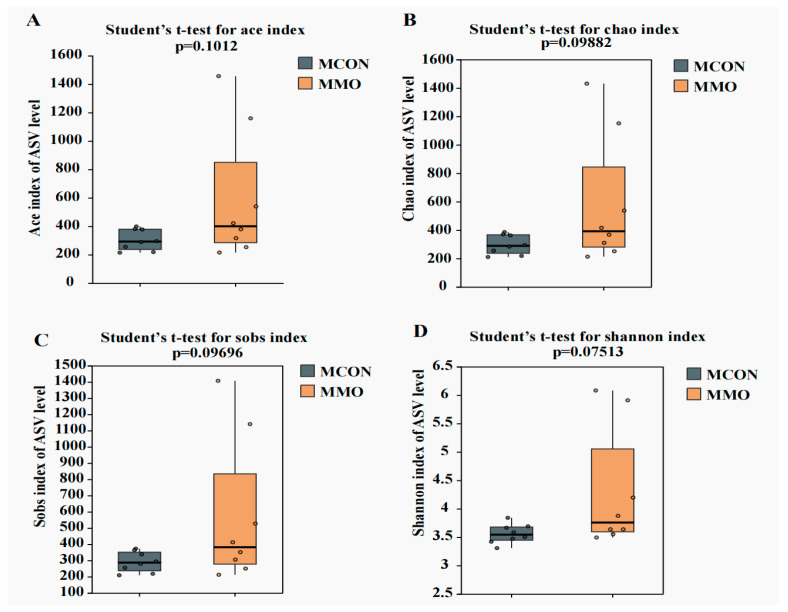
Effects of Mannan Oligosaccharide Supplementation on Alpha Diversity Indices in Dezhou Donkeys. (**A**) Alpha diversity indices (ACE); (**B**) Alpha diversity indices (Chao); (**C**) Alpha diversity indices (Sobs); (**D**) Alpha diversity indices (Shannon). Data are presented as mean ± standard deviation (*n* = 8 per group).

**Figure 6:**
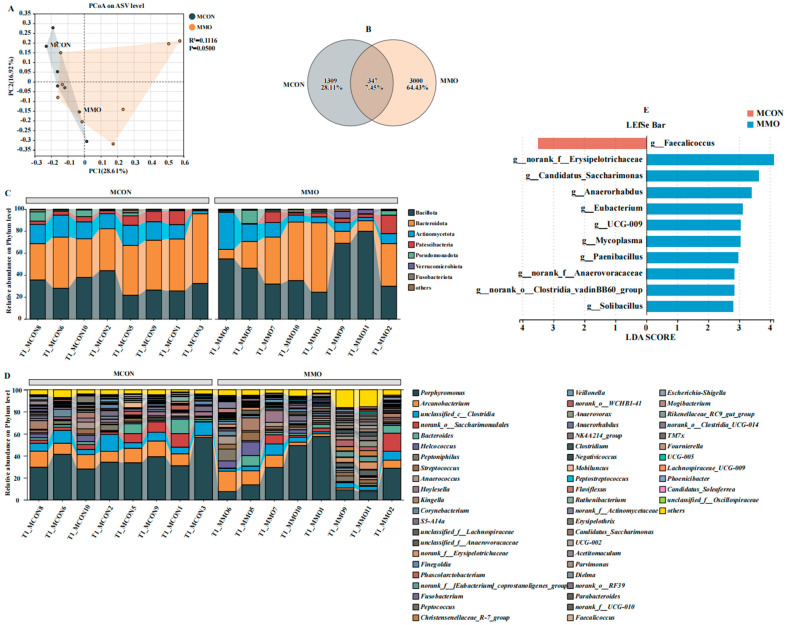
*Effects of Mannan Oligosaccharide Supplementation on Fecal Microbiota in Dezhou Donkeys*. (**A**) Principal Coordinate Analysis (PCoA) based on Bray–Curtis dissimilarity shows beta diversity between the MCON and MMO groups; (**B**) Venn diagram of shared and unique ASVs between groups; (**C**) Microbial community composition at the phylum level; (**D**) Microbial community composition at the genus level; (**E**) LEfSe analysis identifying differentially abundant taxa between groups (LDA score > 2.0, *p* < 0.05). Data are presented as mean ± standard deviation (*n* = 8 per group).

Alpha diversity indices (ACE, Chao1, Sobs, Shannon) were higher in the MMO group, demonstrating enhanced microbial richness and diversity compared to the MCON group. Beta diversity analysis based on PCoA revealed clear separation between the groups, which was further supported by PERMANOVA (R^2^ = 0.1116, *p* = 0.05), indicating that MOS supplementation accounted for approximately 11% of the compositional variation. Venn analysis highlighted distinct ASV profiles, with the MMO group containing a notably higher number of unique ASVs (Figure 5 and Figure 6A,B).

### 2.5. Effects of Mannan Oligosaccharide Supplementation on the Differential Fecal Microbial Community in Dezhou Donkeys

Microbial composition differed markedly between the two groups at both the phylum and genus levels (Figure 6C,D). Major phyla including Bacteroidota, Bacillota, Actinobacteriota, and Patescibacteria showed clear shifts in relative abundance. At the genus level, the MMO group exhibited enrichment of beneficial microbes such as *Clostridium*, *Bacteroides*, *Parabacteroides*, and *Lachnospiraceae_UCG-009*, while *Faecalicoccus* was more dominant in the MCON group. LEfSe analysis identified these differentially abundant taxa as potential microbial biomarkers linked to MOS supplementation (LDA score > 2.0, *p* < 0.05).

## 3. Discussion

Lactation represents a metabolically demanding physiological state in female donkeys, frequently inducing negative energy balance and reduction in body condition [8]. In this study, MOS supplementation markedly attenuated weight loss, suggesting improved nutrient utilization efficiency—a conclusion supported by elevated serum TP and ALB levels. As ALB plays critical roles in maintaining osmotic pressure and transporting nutrients, hormones, and metabolites [3], its increase reflects enhanced nutrient absorption and delivery capacity. Furthermore, MOS supplementation demonstrated hepatoprotective effects, as indicated by reduced ALT levels—a marker of hepatocellular integrity [11]. Moreover, pronounced improvements in lipid metabolism were observed: the MOS group exhibited significantly lower TG and LDL-C, along with elevated HDL-C, which facilitates reverse cholesterol transport and supports cardiovascular health [12,13,14,15]. These results suggest that MOS promotes lipid homeostasis and reduces cardiovascular risk. Additionally, increased TP levels indicate more efficient protein digestion and deposition [16], further underscoring the role of MOS in improving protein metabolism. In summary, dietary MOS enhanced metabolic function in lactating donkeys by improving protein and lipid utilization, supporting hepatic health, and promoting cardiovascular protection. Thus, MOS represents a promising functional feed additive for enhancing metabolic resilience during lactation.

L-4-Chlorotryptophan, a chlorinated tryptophan analog, modulates the kynurenine and serotonin pathways by potentially inhibiting IDO and TPH, reducing neurotoxic metabolites and supporting neuroimmune regulation [17,18]. Its microbial metabolism may yield AhR-activating compounds, further linking gut microbiota to host physiology [19]. In this study, elevated serum levels in MOS-fed donkeys suggest enhanced tryptophan metabolism. Gly-Trp, a glycine-tryptophan dipeptide, improves amino acid availability and gut health. Glycine enhances mucosal integrity and inhibits NLRP3 inflammasome activation [20], while tryptophan-derived metabolites reinforce barrier function via AhR signaling [21,22]. Additionally, glycine facilitates glutathione synthesis and hepatic detoxification, and both glycine and tryptophan contribute to T lymphocyte proliferation and systemic immune modulation [23]. The increased Gly-Trp levels observed in the MOS group reflect improved amino acid utilization and enhanced intestinal protection. Cholylthreonine, a bile acid conjugate, facilitates lipid digestion and intestinal barrier maintenance through enhanced mucin production and immune modulation [24,25,26]. Its elevation in the MOS group indicates improved lipid metabolism and mucosal defense. Collectively, MOS promotes metabolic health in lactating donkeys by enhancing bioactive metabolite production, supporting intestinal barrier function, and regulating immune and antioxidant pathways—contributing to improved nutrient utilization and overall physiological resilience.

The donkey hindgut, particularly the cecum, is a major site of microbial fermentation and plays a central role in nutrient digestion, absorption, and host health [25]. In lactating donkeys, hindgut microbiota and their metabolites are closely linked to nutrient utilization, antioxidant activity, and inflammatory regulation [27]. In this study, MOS supplementation increased alpha diversity indices (ACE, Chao1, Shannon) and induced distinct shifts in beta diversity, indicating a restructured microbial ecosystem. Notably, the MOS group showed selective enrichment of functional taxa, including nutrient-metabolizing genera (e.g., *norank_f__Erysipelotrichaceae*, *Candidatus_Saccharimonas*, *UCG-009*), immunomodulatory genera (e.g., *Eubacterium*, *Anaerorhabdus*, *Mycoplasma*), and gut barrier–supporting taxa (e.g., *Paenibacillus*, *Clostridia_vadinBB60_group*, *Solibacillus*), indicating enhanced metabolic efficiency, immune regulation, and intestinal integrity. Notably, MOS significantly increased the abundance of Bacteroides, a keystone genus in hindgut fermentation that degrades complex carbohydrates into short-chain fatty acids (SCFAs) such as acetate, propionate, and butyrate. These SCFAs are absorbed and utilized in hepatic energy and lipid metabolism [28]. *Bacteroides* also modulates bile acid metabolism via bile salt hydrolases, supporting lipid homeostasis through FXR signaling [29,30]. Additionally, MOS enriched *Lachnospiraceae_UCG-009*, a fiber-degrading genus that produces cellulases and xylanases to break down recalcitrant plant polysaccharides [28]. The resulting SCFAs help activate anti-inflammatory pathways and maintain mucosal barrier function [31]. *Clostridium*, another genus enriched by MOS supplementation, contributes to host health through multiple mechanisms. It facilitates the breakdown of dietary polysaccharides via enzymes such as cellulases, glycosidases, amylases, and proteases, enhancing nutrient absorption and inhibiting pathogens through acid production [32,33]. *Clostridium* also enhances systemic immunity by increasing immunoglobulins (IgG, IgA) and complement proteins (e.g., C4), while its metabolites, including butyrate and hydrogen, activate antioxidant enzymes (e.g., SOD, peroxidase) and reduce oxidative stress via Nrf2 signaling [33,34,35,36,37]. Similarly, MOS increased the abundance of *Parabacteroides*, which participates in bile acid metabolism and anti-inflammatory regulation. This genus converts primary bile acids into secondary forms (e.g., lithocholic acid, ursodeoxycholic acid), promoting lipid homeostasis through FXR activation and enhancing gut barrier integrity [37]. *Parabacteroides* also produces succinate, which supports intestinal gluconeogenesis and glucose balance, and suppresses pro-inflammatory cytokines such as TNF-α, IL-6, and IL-17 [38,39]. Collectively, these findings demonstrate that MOS supplementation enhances hindgut fermentation, enriches beneficial microbial taxa, and improves energy harvesting, metabolic health, and immune function in lactating donkeys. These benefits position MOS as a promising prebiotic for supporting sustainable donkey production.

## 4. Materials and Methods

### 4.1. Experimental Design

Sixteen healthy lactating Dezhou jennies (age: 2–3 years; body weight: 269.88 ± 31.84 kg) in uniform condition, along with their corresponding foals, were included in the study. All animals were maintained under standardized breeding conditions at Shandong Dong’e Ejiao Co., Ltd. (Liaocheng, China). Jennies were housed individually in semi-open pens and received a standard farm-formulated diet provided ad libitum twice daily (07:00 and 19:00), with free access to clean drinking water. No probiotics or antibiotics had been administered for at least three months prior to or during the trial. A three-day adaptation period preceded the experiment to stabilize feed intake. After acclimation, the jennies were weighed and randomly assigned to two treatment groups (n = 8 per group). The trial lasted 60 days. The control group (MCON) received the basal diet (as outlined in Table 1) without MOS supplementation, while the MMO group was fed the same basal diet supplemented with 0.5 g/kg of mannan oligosaccharide (Actigen™, Beijing Alltech Biological Products Co., Ltd., Beijing, China). Throughout the experiment, growth performance and diarrhea incidence were regularly monitored and recorded for both jennies and foals.

### 4.2. Sample Collection

On the morning of the final day of the formal trial, both the female donkeys (jennies) and their foals were weighed on an empty stomach prior to feeding. Blood samples (5 mL) were collected from the jugular vein of each animal into sterile tubes before the morning feeding. Serum was separated by centrifugation at 3000× *g* for 10 min at 4 °C, then immediately snap-frozen in liquid nitrogen and stored for subsequent analysis. Fecal samples were collected aseptically from the perianal region. All samples were handled under sterile conditions to prevent contamination, transferred into sterile 2 mL centrifuge tubes, immediately frozen in liquid nitrogen, and transported on dry ice to the laboratory. Upon arrival, they were stored at −80 °C until analysis.

### 4.3. Serum Biochemical Indices

Serum biochemical parameters, including alanine aminotransferase (ALT, C009-2-1), aspartate aminotransferase (AST, C010-2-1), alkaline phosphatase (ALP, A059-2-2), total protein (TP, A045-2-2), albumin (ALB, A028-2-1), total cholesterol (TC, A111-1-1), triglycerides (TG, A110-1-1), high-density lipoprotein cholesterol (HDL-C, A112-1-1), and low-density lipoprotein cholesterol (LDL-C, A113-1-1), were analyzed using a fully automated biochemical analyzer (Jiancheng Bioengineering Institute, Nanjing, China) in accordance with the manufacturer’s protocols. Each treatment group consisted of eight biological replicates, with each replicate measured in two technical replicates.

### 4.4. Plasma Metabolomics Analysis

Metabolomic analysis of plasma samples was conducted to characterize the systemic metabolic alterations induced by dietary MOS supplementation in lactating donkeys. Untargeted metabolomic profiling was carried out using ultra-high-performance liquid chromatography coupled with a Fourier transform quadrupole-orbitrap mass spectrometer (UHPLC-Q Exactive HF-X, Thermo Fisher Scientific, Waltham, MA, USA), enabling comprehensive detection of diverse metabolite classes. Chromatographic separation was achieved on an HSS T3 column (100 mm × 2.1 mm, 1.8 µm; Waters, Waltham, MA, USA), selected for its superior retention and resolution characteristics. Sample preparation included protein precipitation and metabolite extraction using high-purity chromatographic solvents, supported by standard instrumentation including a nitrogen evaporator, centrifugal concentrator, homogenizer, ultrasonic cleaner, refrigerated centrifuge, and analytical balance. To ensure data reproducibility and monitor analytical variability, pooled quality control (QC) samples were regularly inserted throughout the analytical sequence, allowing continuous evaluation of system performance and measurement stability.

### 4.5. Microbiota Sequencing and Analysis

Total genomic DNA was extracted from 200 mg of each fecal sample using the E.Z.N.A.^®^ Soil DNA Kit (Omega Bio-tek, Norcross, GA, USA) in accordance with the manufacturer’s instructions. The V3–V4 hypervariable regions of the bacterial 16S rRNA gene were amplified with the universal primers 338F and 806R. The resulting PCR products were examined by 2% agarose gel electrophoresis, purified using the AxyPrep DNA Gel Recovery Kit (Axygen Biosciences, Union City, CA, USA), and quantified with a Qubit 2.0 Fluorometer (Thermo Fisher Scientific, Waltham, MA, USA). Equimolar amounts of the purified amplicons were pooled and subjected to paired-end sequencing (2 × 250 bp) on an Illumina MiSeq platform (Illumina, San Diego, CA, USA), following the standard protocols provided by Majorbio Bio-Pharm Technology Co., Ltd. (Shanghai, China).

### 4.6. Statistical Analysis

All data are expressed as mean ± standard deviation (SD). Statistical differences between the two groups were evaluated using a two-tailed unpaired Student’s *t*-test. For multiple comparisons, Duncan’s multiple range test was applied. Data visualization and statistical analyses were conducted using GraphPad Prism version 9.5 (GraphPad Software, San Diego, CA, USA). Statistical significance was set at *p* < 0.05, with *p* < 0.01 and *p* < 0.001 indicating increasingly significant differences.

Raw LC-MS data were initially processed with Progenesis QI (Waters Corporation, Milford, MA, USA) to perform baseline correction, peak detection, integration, retention time alignment, and normalization. Subsequently, a comprehensive data matrix containing retention time, *m*/*z*, and peak intensity was generated for further statistical analysis. Multivariate statistical analyses, including principal component analysis (PCA) and orthogonal partial least squares discriminant analysis (OPLS-DA), were then carried out using SIMCA (version 14.1, Umetrics, Umeå, Sweden). To ensure model reliability, its validity was carefully evaluated based on R^2^ and Q^2^ values and further validated through 200 permutation tests, thereby minimizing the risk of overfitting. For the identification of differential metabolites, variable importance in projection (VIP) scores greater than 1.0 and *p*-values less than 0.05 were applied as criteria, using either Student’s *t*-test or the Wilcoxon rank-sum test. Afterwards, metabolite annotation was conducted by matching acquired MS and MS/MS spectra against the Human Metabolome Database (HMDB). Finally, to gain biological insight into the altered metabolites, KEGG pathway enrichment analysis was performed with MetaboAnalyst 5.0 and KEGG Mapper. This integrative approach facilitated the identification of significantly perturbed metabolic pathways and provided mechanistic insights into the regulatory effects of MOS supplementation.

Raw sequencing data were processed in QIIME2 for quality control, denoising, merging, and chimera removal, resulting in a minimum of 50,000 high-quality reads per sample. Subsequently, amplicon sequence variants (ASVs) were identified with the DADA2 algorithm and further taxonomically classified using the RDP Classifier (v2.2) against the SILVA database (release 138). Alpha diversity indices—including ACE, Chao1, Shannon, and Sobs—were calculated in QIIME2 and with the vegan package (v2.5.6) in R. Additionally, a Venn diagram was generated using the VennDiagram package (v3.1.1) to visualize shared and unique ASVs across groups. For beta diversity analysis, principal coordinate analysis (PCoA) was performed based on Bray–Curtis distances. Furthermore, statistical significance of group differences was evaluated with permutational multivariate analysis of variance (Adonis) in R (v3.2.1). To compare microbial composition at the genus level, the vegan package (v3.5.1) was applied. Specifically, the Wilcoxon rank-sum test and Kruskal–Wallis test were used for statistical comparisons, followed by pairwise comparisons where appropriate. In order to identify differentially abundant taxa, linear discriminant analysis effect size (LEfSe) was employed, with an LDA score threshold of >2.0 and a *p*-value < 0.05. Finally, functional prediction of microbial communities was performed with PICRUSt2 (v2.5.0). The resulting predictions were annotated against the Kyoto Encyclopedia of Genes and Genomes (KEGG) Orthology database, thereby enabling inference of microbial functional potential and ecological relevance from 16S rRNA gene data.

## 5. Conclusions

Dietary supplementation with mannan oligosaccharides (0.5 g/kg) improved metabolic health and gut microbiota composition in lactating Dezhou donkeys. Specifically, MOS enhanced serum lipid profiles and up-regulated pathways related to anti-inflammatory, antioxidant, and immunomodulatory functions. Furthermore, it increased beneficial bacteria such as *Bacteroides* and *Clostridium*, which support fiber digestion and gut barrier integrity. Although final body weight remained unaffected, MOS supplementation alleviated lactational weight loss and improved metabolic and microbial parameters, thereby contributing to overall lactational resilience and potential milk quality enhancement.

## Figures and Tables

**Table 1:** Composition and nutritional level of experimental concentrate (dry-matter basis).

Ingredients	Content (%)	Nutrients	Nutrient Level (%)
Corn	31.75	Dry matter	88.53
Middlings bran	12.00	Crude protein	17.18
Wheat flour middling	21.00	Ash	8.37
DDGS	5.00	Crude fiber	4.27
Wheat bran	18.00	Ether extract	4.59
Soybean meal	6.65	Calcium	1.27
NaCl	0.63	Phosphorus	0.52
CaCO_3_	3.72	Lysine	0.75
CaHPO_4_	0.25		
Premix1	1.00		
Total	100.00		

Premix/kg: VA, 600 KIU; VD_3_, 125 KIU; VE, 3500 IU; Fe, 2 g; Cu, 800 mg; Zn, 6 g; Mn, 13 g; I, 95 mg; Se, 50 mg.

## Data Availability

The original contributions generated for this study are included in the article; further inquiries can be directed to the corresponding author.

## Abbreviations

The following abbreviations are used in this manuscript:

ACE	Alpha diversity indices
AhR	aryl hydrocarbon receptor
ABCA1	ATP-binding cassette sub-family A member 1
ABCG1	ATP-binding cassette sub-family G member 1
ASV	Amplicon Sequence Variant
ALT	alanine aminotransferase
AST	aspartate aminotransferase
ALP	alkaline phosphatase
ALB	albumin
ADG	average daily gain
BI	Class B Type I
CD4+	Cluster of Differentiation 4-positive T lymphocyte
CD8+	Cluster of Differentiation 8-positive T lymphocyte
CA	cholic acid
DA	the Differential Abundance
FCR	feed conversion ratio
FXR	Farnesoid X Receptor
GSH	glutathione
Gly-Trp	Glycyl-Tryptophan
HDL	High-Density Lipoprotein
HMDB	The Human Metabolome Database
LDL	Low-Density Lipoprotein
IDO	Indoleamine 2,3-Dioxygenase
I3P	indole-3-pyruvate
IPA	indolepropionic acid
IL-1β	Interleukin-1 beta
IL-8	Interleukin-8
IL-6	Interleukin-6
IBD	inflammatory bowel disease
IgY	immunoglobulin Y
IgM	immunoglobulin M
LPS	lipopolysaccharide
LDA	Linear Discriminant Analysis
MMO	the mannan oligosaccharide-treated group
MUC2	Mucin 2
MCON	the not MOS-supplemented group
MOS	Mannan oligosaccharides
NADH	Nicotinamide Adenine Dinucleotide
NADPH	Nicotinamide Adenine Dinucleotide Phosphate
OPLS-DA	orthogonal partial least squares discriminant analysis
PCA	principal component analysis
PCoA	principal coordinate analysis
PC1	Principal Component 1
PLA2	pancreatic lipase
pTreg	peripheral T regulatory
QC	quality control
RCT	reverse cholesterol transport
SOD	Superoxide Dismutase
SD	standard deviation
TNF-α	Tumor Necrosis Factor-alpha
TC	total cholesterol
TG	total triglyceride
TPH	tryptophan hydroxylase
TP	total protein
Thr	threonine
TCA	tricarboxylic acid
VFAs	volatile fatty acids
VIP	Variable Importance in Projection
